# Adaptation of the Invasive Plant *Sphagneticola trilobata* (L.) Pruski to Drought Stress

**DOI:** 10.3390/plants13162207

**Published:** 2024-08-09

**Authors:** Qilei Zhang, Ye Wang, Zhilong Weng, Guangxin Chen, Changlian Peng

**Affiliations:** 1 Guangzhou Key Laboratory of Subtropical Biodiversity and Biomonitoring, Guangdong Provincial Key Laboratory of Biotechnology for Plant Development, School of Life Sciences, South China Normal University, Guangzhou 510631, China; 2 Research Institute of Tropical Forestry, Chinese Academy of Forestry, Guangzhou 510520, China

**Keywords:** PEG-6000, *Sphagneticola trilobata*, hybrid, biological invasion, abscisic acid

## Abstract

Invasive species and their hybrids with native species threaten biodiversity. However, there are few reports on the drought stress adaptability of invasive species *Sphagneticola trilobata* (L.) Pruski and its hybrid with native species *S. calendulacea*. In this study, relative water content (RWC), abscisic acid (ABA), reactive oxygen species, antioxidant capacity, and photosynthetic capacity were measured in the hybrid and its parents under drought stress (13% PEG-6000). Under drought stress, the ABA content and RWC in *S. trilobata* were the highest. RWC decreased by 28% in *S. trilobata*, 41% in *S. calendulacea*, and 33% in the hybrid. Activities of the antioxidant enzymes in *S. trilobata* were the highest, and the accumulation of malondialdehyde (MDA) was the lowest (4.3 μg g^−1^), while it was the highest in *S. calendulacea* (6.9 μg g^−1^). The maximum photochemical efficiency (F_v_/F_m_) of *S. calendulacea* was the lowest (0.71), and it was the highest in *S. trilobata* (7.5) at 8 h under drought stress. The results suggest that the drought resistance of the hybrid was weaker than that of *S. trilobata* but stronger than that of *S. calendulacea*. Therefore, the survival of *S. calendulacea* may be threatened by both the invasive species *S. trilobata* and the hybrid.

## 1. Introduction

Biological invasions are one of the primary issues in ecology [1,2,3]. Invading organisms may hybridize with native species in the invaded area during the invasion process, forming hybrid species. Currently, about 25% of invasive plants have hybridized with native species [4]. The adaptability of the hybrid species produced between invasive and native species is stronger than that of their parents, and the range of adaptation is wider in the invaded area [5,6]. The diversity of native species is reduced by invasive species and their hybrids with native species, thus posing a threat to endangered species [7].

With global warming, extremely harsh environments, such as drought environments, are becoming more frequent and severe [8]. Drought stress is one of the most common abiotic stresses detrimental to plant growth and development [9]. Due to the lack of water in plants, cells lose tension, leaves wilt, tender parts droop, leaf thickness increases, leaf area decreases, the plant becomes shorter, the root system lengthens, the root–shoot ratio increases, etc. [9,10,11,12]. In addition, as the basic medium of physiological reactions in plants, a shortage of water will seriously affect various physiological reactions in cells [13]. Excessive reactive oxygen species (ROS) are generated and accumulate in plant cells due to the oxidative stress caused by drought stress. High concentrations of ROS may attack the biological macromolecules in cells, including lipids, proteins, and nucleic acids [14]. Excessive ROS disrupt the stability of cell membranes, leading to membrane lipid peroxidation, as well as increasing the membrane permeability of malondialdehyde (MDA) content in cells. The function of selective passage through the cell membrane is lost due to the destruction of the cell membrane. The inability to effectively control the entry and exit of substances into cells results in them being unable to perform normal metabolic activities [15,16].

In order to reduce the damage from drought stress, some plants have evolved strategies to cope with it. Leaf stomata are the main channels for water loss in terrestrial plants, and the aperture directly affects the rate of water loss. Reducing the leaf stomatal opening effectively reduces water loss [13]. Abscisic acid (ABA) plays an important role in regulating the stomatal opening. The content of ABA and the expression of genes related to ABA synthesis are significantly upregulated under drought stress [13]. In addition, osmotic regulators, such as soluble sugar and proline, accumulate in plants to maintain the osmotic pressure of cells under drought stress [17]. Furthermore, to eliminate excessive ROS in cells, the activities of antioxidant enzymes and the contents of non-enzymatic antioxidant substances in cells increase [18,19]. In a study on the invasive plant *Alternanthera philoxeroides* (Mart.) Griseb and the model plant maize (*Zea mays* L.), it was found that drought stress increased the content of ROS. With the increase in the content of ROS, drought stress significantly increased the antioxidant enzyme activity in *A*. *philoxeroides* and maize, and the content of antioxidant substances also significantly increased [18,20].

*Sphagneticola trilobata* (L.) Pruski (synonym: *Wedelia trilobata* (L.) Hitchc.), an invasive species in southern China, is a perennial herbaceous plant in the Asteraceae family, and it is native to South and Central America. However, in the field, researchers have discovered a hybrid between the invasive species *S. trilobata* and the native species *S. calendulacea* [21]. Previous studies have shown that the tolerance of the hybrid to low temperatures and nitrogen deposition was between that of its two parents [22,23]. However, the tolerance of the hybrid to cadmium stress was higher than that of its two parents in our recent report [6], and the tolerance of the hybrid to flooding stress was higher than that of its invasive parent [24]. However, there have been few reports on whether this hybrid shows heterosis in tolerance to drought stress.

In this study, the responses of the hybrid and its parents (*S. trilobata* and *S. calendulacea*) to drought stress were compared. We aimed to investigate the tolerance of the invasive species *S. trilobata* and the hybrid to drought stress and explore whether the hybrid exhibits a super-parental advantage under drought stress.

## 2. Results

### 2.1. Phenotypic Characteristics

**Figure 1:**
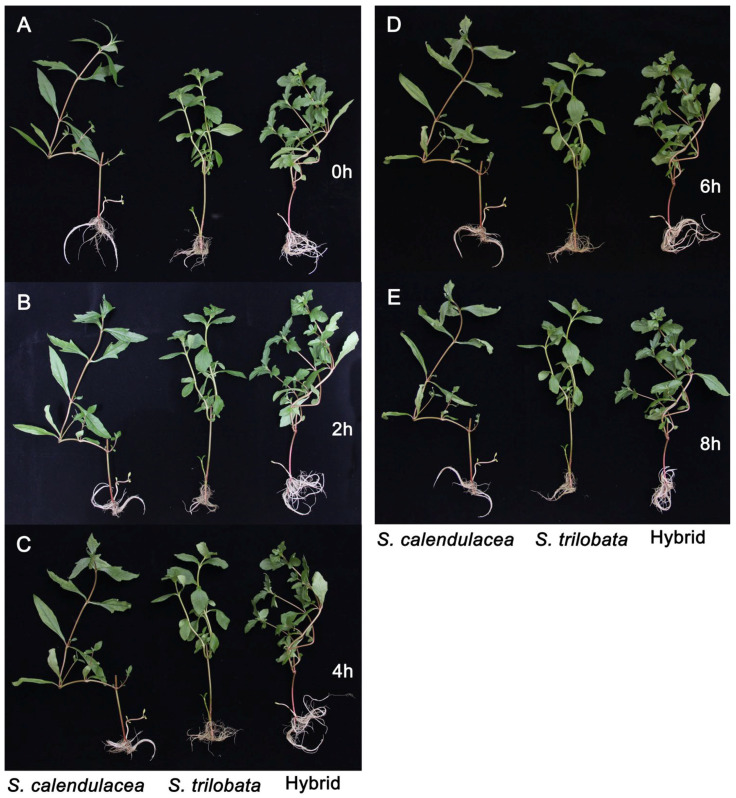
Leaf phenotypic changes in *S. calendulacea*, *S. trilobata,* and their hybrid at 0 h (0 h, **A**), 2 h (2 h, **B**), 4 h (4 h, **C**), 6 h (6 h, **D**), and 8 h (8 h, **E**) under PEG-6000-simulated drought stress.

Under osmotic stress (13% PEG-6000), the leaves of the parents and hybrid wilted over time. However, the responses of the three species to the PEG-6000 treatment differed. Among them, the leaves of the native species *S. calendulacea* wilted first, after PEG-6000 treatment for 2 h (Figure 1B). After treatment for 6 h, the edges of the *S. calendulacea* leaves became necrotic due to excessive water loss (Figure 1D). The results show that the invasive species *S. trilobata* and its hybrid only wilted during the 8 h treatment with PEG-6000.

### 2.2. Leaf Water and ABA Content, as Well as Expression of Related Genes

**Figure 2:**
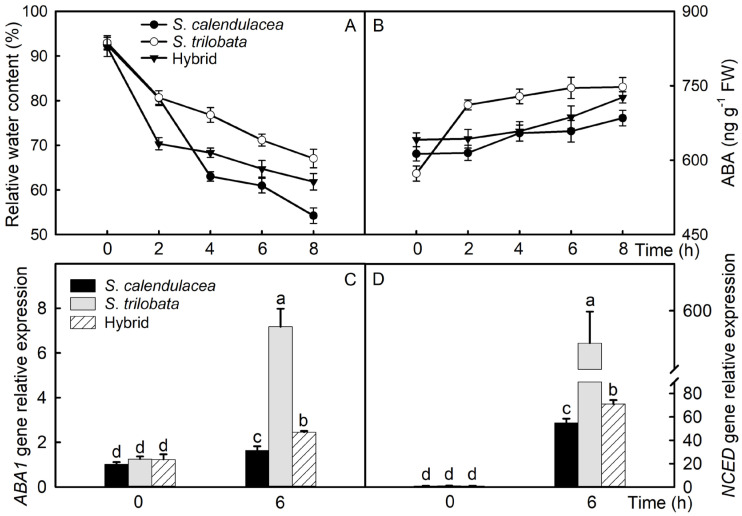
Under PEG-6000-simulated drought stress, the changes in relative water content (**A**), abscisic acid (ABA, **B**) content, relative expression of the zeaxanthin-epoxidase gene (*ABA1*, **C**), and 9-cis-epoxycarotenoid dioxygenase gene (*NCED*, **D**) in the leaves of *S. calendulacea*, *S. trilobata,* and their hybrid. FW, fresh weight. Five biological replicates. Above bars, different lowercase letters indicate statistical significance (*p* < 0.05).

Under the PEG-6000 treatment, the relative water content in the leaves of the hybrid and its parents, *S. calendulacea* and *S. trilobata,* decreased gradually. Among them, the *S. calendulacea* leaves showed the fastest water loss rate and the lowest water content, the *S. trilobata* leaves showed the slowest water loss rate and the highest water content, and the hybrid’s leaves showed intermediate results between its parents (Figure 2A). The relative water content was 61% in the hybrid, 54% in *S. calendulacea*, and 67% in *S. trilobata*, and it decreased by 33%, 41%, and 28%, respectively, at 8 h under drought stress. ABA plays a key role in controlling leaf water loss. In this experiment, the ABA content in the leaves of the hybrid and its parents, *S. calendulacea* and *S. trilobata,* accumulated with the decrease in water content, and it increased gradually. The leaves of *S. trilobata* showed the fastest and highest increase in ABA content, the leaves of *S. calendulacea* showed the slowest and lowest increase in ABA content, while the leaves of the hybrid showed intermediate results between its parents (Figure 2B). The genes of zeaxanthin-epoxidase (*ABA1*) and 9-cis-epoxycarotenoid dioxygenase (*NCED*) are associated with ABA synthesis, and the PEG-6000 treatment significantly increased their relative expression levels. Under the PEG-6000 treatment, the gene expression levels of *ABA1* and *NCED* in the leaves of *S. trilobata* were significantly higher than in the hybrid. They were the lowest in the leaves of *S. calendulacea* (Figure 2C,D). The relative expression level of *ABA1* increased by 62% in *S. calendulacea*, 473% in *S. trilobata*, and 99% in the hybrid at 6 h under drought stress. The relative expression level of *NCED* increased 54 times in *S. calendulacea*, 555 times in *S. trilobata*, and 71 times in the hybrid at 6 h under drought stress.

### 2.3. Leaf Stomata, Proline, and Soluble Sugar

**Figure 3:**
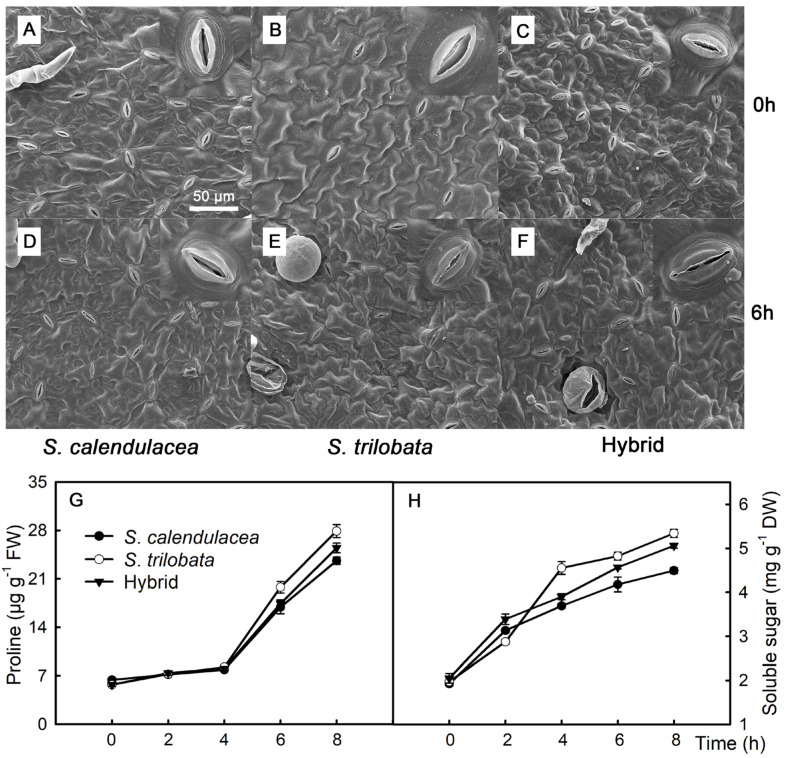
Under PEG-6000-simulated drought stress, the changes in leaf stomatal size (**A**–**F**), proline (**G**), and soluble sugar (**H**) content in leaves of the hybrid and its parents *S. calendulacea* and *S. trilobata*. FW, fresh weight; DW, dry weight. Five biological replicates.

Leaf stomata are the main channels for water loss in terrestrial plants, and the aperture directly affects the rate of water loss in plants. Under the PEG-6000 treatment, the leaf stomata opening decreased. It was the smallest in *S. trilobata* and the largest in *S. calendulacea* (Figure 3A–F). Proline and soluble sugar are the main osmotic regulators in plant leaves, and they regulate the osmotic pressure. During the PEG-6000 treatment, the proline and soluble sugar contents in the leaves of the hybrid and its parents increased gradually. The proline content increased slowly during the first 0–4 h of treatment and faster from 4 to 8 h. During the 4–8 h of treatment, the proline and soluble sugar contents in the leaves of *S. trilobata* increased faster than in *S. calendulacea* and its hybrid, and they were also higher in the leaves of *S. trilobata* than in the hybrid and *S. calendulacea*. The proline and soluble sugar contents were the lowest in *S. calendulacea* (Figure 3G,H). The proline content was 23.6 μg g^−1^ in *S. calendulacea*, 27.9 μg g^−1^ in *S. trilobata*, and 25.5 μg g^−1^ in the hybrid at 8 h under drought stress. The soluble sugar content was 4.5 mg g^−1^ in *S. calendulacea*, 5.3 mg g^−1^ in *S. trilobata*, and 5.1 mg g^−1^ in the hybrid at 8 h under drought stress.

### 2.4. Hydrogen Peroxide, Superoxide Anion, MDA, and Enzyme Activity

**Figure 4:**
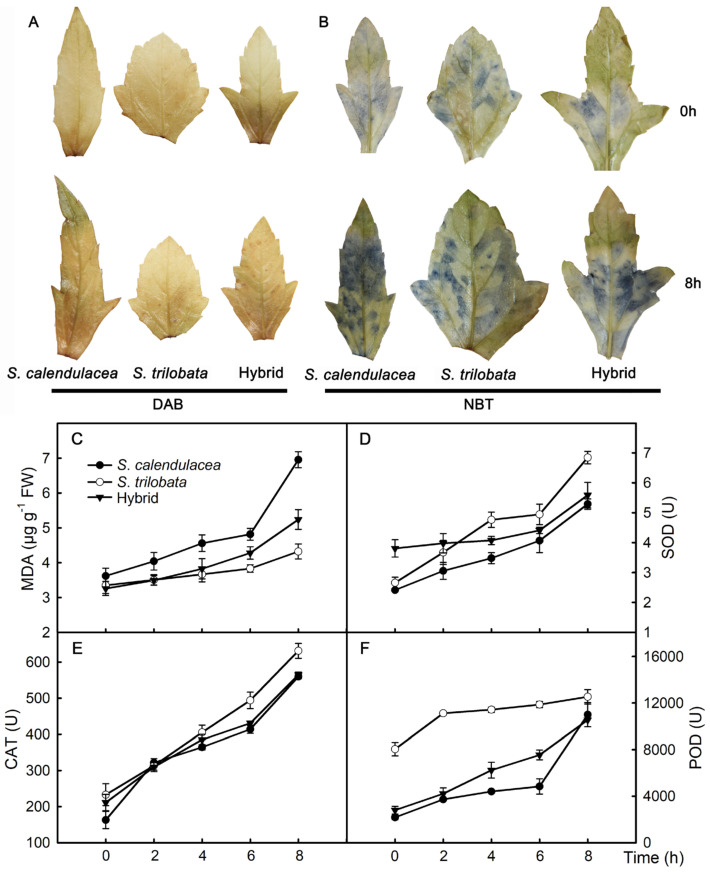
Under PEG-6000-simulated drought stress, the accumulation of hydrogen peroxide (DAB staining, **A**), superoxide anion (NBT staining, **B**), the changes in content of malondialdehyde (MDA, **C**), and activities of superoxide dismutase (SOD, **D**), catalase (CAT, **E**), and peroxidase (POD, **F**) in leaves of the hybrid and its parents *S. calendulacea* and *S. trilobata*. DAB: 3,3′-Diaminobenzidine; NBT: nitroblue tetrazolium. FW, fresh weight. Five biological replicates.

After the PEG-6000 treatment, the accumulation of hydrogen peroxide and superoxide anion in the leaves of the hybrid and its parents increased, and the accumulation of superoxide anion in the leaves of the native species *S. calendulacea* was the largest (Figure 4A,B). The MDA content in the leaves of the hybrid and its parents (*S. calendulacea* and *S. trilobata*) increased gradually under the PEG-6000 treatment. The MDA content was 6.9 μg g^−1^ in *S. calendulacea*, 4.3 μg g^−1^ in *S. trilobata*, and 5.2 μg g^−1^ in the hybrid at 8 h; it was the highest in *S. calendulacea* and the lowest in *S. trilobata*, and the hybrid showed intermediate results between its two parents (Figure 4C). To alleviate oxidative stress, antioxidant enzyme activities increase. The superoxide dismutase (SOD), catalase (CAT), and peroxidase (POD) activities in the leaves of the hybrid and its parents gradually increased under the PEG-6000 treatment. During the 4–8 h of treatment, the enzyme activity was the highest in *S. trilobata* and the lowest in *S. trilobata*, and the hybrid showed intermediate results between its two parents (Figure 4D–F). The SOD activity was 5.3 U in *S. calendulacea*, 6.8 U in *S. trilobata*, and 5.6 U in the hybrid at 8 h. The CAT activity was 559 U in *S. calendulacea*, 631 U in *S. trilobata*, and 564 U in the hybrid at 8 h. The POD activity was 10,994 U in *S. calendulacea*, 12,535 U in *S. trilobata*, and 10,542 U in the hybrid at 8 h.

### 2.5. Chlorophyll Fluorescence Parameters and Gas Exchange Parameters

**Figure 5:**
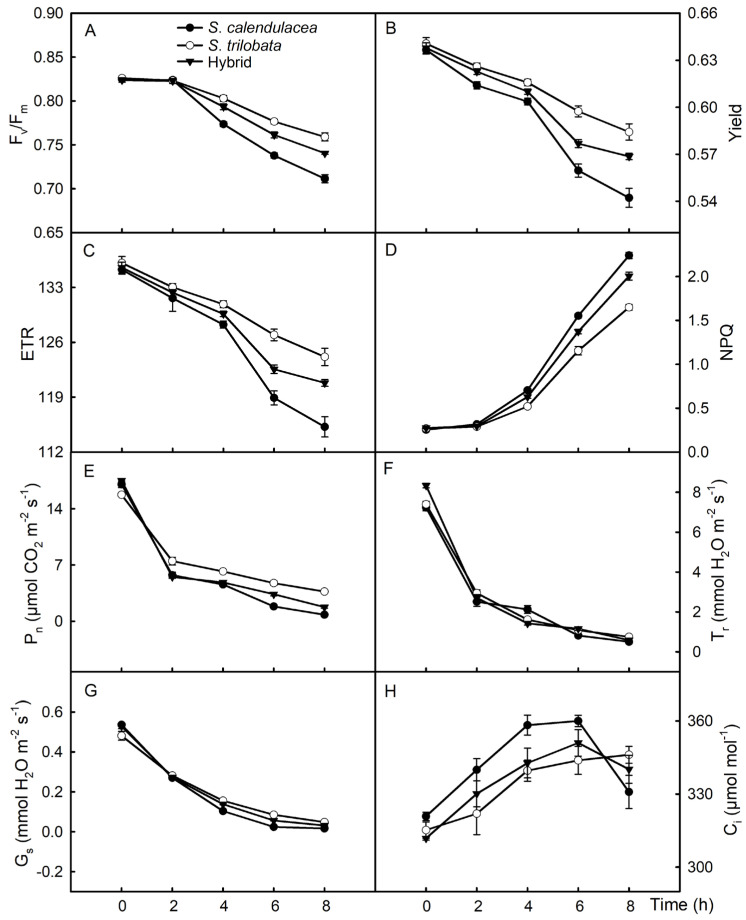
Under PEG-6000-simulated drought stress, the changes in maximum photochemical efficiency (F_v_/F_m_, **A**), actual photochemical efficiency (yield, **B**), electron transport rate (ETR, **C**), non-photochemical quenching (NPQ, **D**), net photosynthetic rate (P_n_, **E**), transpiration rate (T_r_, **F**), stomatal conductance (G_s_, **G**), and intercellular CO_2_ content (C_i_, **H**) in leaves of the hybrid and its parents *S. calendulacea* and *S. trilobata*. Five biological replicates.

During the PEG-6000 treatment, the maximum photochemical efficiency (F_v_/F_m_) of the hybrid and its parents (*S. calendulacea* and *S. trilobata*) decreased gradually (Figure 5A). However, the decline rate of F_v_/F_m_ differed among the three species. The decline rate of F_v_/F_m_ was the slowest in *S. trilobata*, and its value was higher than in the hybrid and *S. calendulacea* during the 4–6 h of treatment, while *S. calendulacea* showed the fastest decline rate and the lowest F_v_/F_m_ value. The F_v_/F_m_ value was 7.1 in *S. calendulacea*, 7.5 in *S. trilobata*, and 7.4 in the hybrid at 8 h. The variation trend of the actual photochemical efficiency (yield) and electron transport rate (ETR) was consistent with that of F_v_/F_m_ (Figure 5B,C). In addition, the variation trend of non-photochemical quenching (NPQ) was the opposite of that of F_v_/F_m_, showing a gradual increase in the hybrid and its parents under the PEG-6000 treatment (Figure 5D). NPQ was the slowest in *S. trilobata* and lower than that of the native species *S. calendulacea* and the hybrid, while it was the fastest in *S. calendulacea*, which showed the highest NPQ.

Photosynthesis is the basis of plant growth, and a highly efficient photosynthetic rate is beneficial for plant growth. In the process of the PEG-6000 treatment, the net photosynthetic rate (P_n_) of the hybrid and its parents decreased gradually. P_n_ decreased faster during the first 0–2 h of treatment, while the decrease rate was slower in the 2–8 h of treatment (Figure 5E). P_n_ was higher in the invasive species *S. trilobata* than in the hybrid and *S. calendulacea*, while it was higher in the hybrid than in *S. calendulacea* after the PEG-6000 treatment for 4 h. P_n_ was 0.8 μmol m^−2^ s^−1^ in *S. calendulacea*, 3.7 μmol m^−2^ s^−1^ in *S. trilobata*, and 1.8 μmol m^−2^ s^−1^ in the hybrid at 8 h. The change trends of the stomatal conductance (G_s_) and transpiration rate (T_r_) were consistent and decreased gradually during the PEG-6000 drought stress treatment (Figure 5F,G). During the 0–8 h of the PEG-6000 treatment, the change in the intercellular CO_2_ (C_i_) content showed a trend of first increasing and then decreasing in the hybrid and *S. calendulacea*, while it gradually increased in the leaves of *S. trilobata* (Figure 5H).

## 3. Discussion

Under 13% PEG-6000-simulated drought stress, the leaf relative water content decreased the slowest in *S. trilobata* and decreased the fastest in *S. calendulacea*, and the hybrid showed intermediate results between its two parents (Figure 2A). The leaves wilted quicker in *S. calendulacea* than in the hybrid and *S. trilobata*, and the edges of the leaves curled after 4 h of the 13% PEG-6000 treatment (Figure 1C). Excessive ROS are generated and accumulate in plant cells due to the oxidative stress caused by drought stress [25]. High concentrations of ROS may attack the biological macromolecules in cells, leading to membrane lipid peroxidation, increased membrane permeability, and leaf damage [26]. Studies have found that drought stress significantly increases the ROS accumulation in sunflower (*Helianthus annuus* L.) leaves, and the more severe the water deficiency, the more ROS accumulate [27]. In this study, the results showed that the ROS content in the leaves of both parents and the hybrid increased under the 13% PEG-6000 treatment. The ROS content was the highest in the native species *S. calendulacea*, while it was the lowest in the invasive species *S. trilobata* (Figure 4A,B). This indicates that *S. calendulacea* suffered the most severe damage under drought stress [27]. Previous studies on *S. trilobata* and *S. calendulacea* found that cell membrane permeability [28] and MDA content [29] increased under stress conditions. This experiment found that the MDA content in the leaves of the three species increased (Figure 4C), indicating that the leaves of the hybrid and its parents were damaged under the 13% PEG-6000 treatment. The MDA content was the highest in the native species *S. calendulacea*, and it was lower in the invasive species *S. trilobata* and the hybrid after 6 h, which indicates that the leaf damage in the native species *S. calendulacea* was the most serious [24]. Huang et al. [25] found that the tolerance of *S. trilobata* was stronger than that of *S. calendulacea* under drought stress and that the MDA content was lower in *S. trilobata*. These results suggest that the tolerance of *S. trilobata* and the hybrid is stronger than that of *S. calendulacea* under drought stress.

Under normal conditions, F_v_/F_m_ does not change significantly. However, F_v_/F_m_ decreases significantly under stress [30,31]. Song et al. [28] found that, under high-temperature stress, the F_v_/F_m_ of *S. trilobata* and *S. calendulacea* decreased, while *S. trilobata* was more tolerant to high temperatures, showing a higher F_v_/F_m_ value. In this study, the results showed that the F_v_/F_m_ values of the hybrid and its parents decreased gradually under drought stress, which is consistent with previous research results [32]. Among them, the F_v_/F_m_ values were the lowest in the native species *S. calendulacea*, while they were the highest in the invasive species *S. trilobata* after 4 h of the 13% PEG-6000 treatment (Figure 5A). The F_v_/F_m_ values were higher in the hybrid than in the native species *S. calendulacea*. The results indicate that drought stress caused the greatest damage to the native species *S. calendulacea* and the least damage to the invasive species *S. trilobata* [28]. In addition, a decrease in water content hinders the metabolic process of cells in leaves [33]. Photosynthesis is the basis of the material accumulation and growth of higher plants. Under drought stress, the photosynthesis of plant leaves is affected, leading to a significant decrease in P_n_ [34], and the results of this study are consistent with this. In this experiment, P_n_ in the leaves of the three species decreased gradually under the 13% PEG-6000 treatment (Figure 5E). P_n_ was highest in the *S. trilobata* leaves and lowest in the *S. calendulacea* leaves, with the hybrid showing intermediate results between its two parents, which was consistent with the change trend in leaf relative water content.

To improve resistance, the stomatal opening of leaves is reduced under drought stress, thereby reducing the rate of water loss through leaf stomata [13,35]. Previous research has shown that applying appropriate concentrations of ABA to plants or increasing their ABA content through gene editing reduces the leaf stomatal opening [36,37]. In this study, the results showed that the ABA content in the hybrid and its parents, *S. calendulacea* and *S. trilobata,* increased under the 13% PEG-6000 treatment. The ABA content was higher in the *S. trilobata* and hybrid leaves than in *S. calendulacea* after 6 h. This indicates that *S. trilobata* and the hybrid rapidly synthesize more ABA to reduce water loss under drought stress [27]. Furthermore, drought stress significantly increases the proline and soluble sugar contents in plants [27,38]. These osmotic regulators not only contribute to osmotic regulation but also protect the structure of biomolecules and membranes, or they act as free radical scavengers to protect deoxyribonucleic acid from ROS damage [39]. In this study, the proline and soluble sugar contents in the hybrid and its parents increased under the 13% PEG-6000 treatment. The proline and soluble sugar contents were higher in *S. trilobata* than in *S. calendulacea*, and they were lower in *S. calendulacea* than in the hybrid after 4 h of the 13% PEG-6000 treatment (Figure 3G,H). This indicates that *S. trilobata* and the hybrid can synthesize and accumulate more proline under drought stress and maintain cell osmotic pressure [39]. In addition, to eliminate excessive ROS in plant cells, antioxidant enzyme activities increase under drought tress [19]. In this experiment, CAT, SOD, and POD activities increased in the hybrid, *S. calendulacea*, and *S. trilobata* under the 13% PEG-6000 treatment. These activities were strongest in *S. trilobata* and weakest in *S. calendulacea*, with the hybrid showing intermediate results between its parents (Figure 4D–F). Previous studies have shown that high antioxidant enzyme activities significantly reduce the ROS content [6]. Huang et al. [29] found that the antioxidant defense system in *S. trilobata* was stronger than that in *S. calendulacea* under stress conditions. These results indicate that the antioxidant capacity of *S. trilobata* and the hybrid is stronger than that of *S. calendulacea* under drought stress. However, this study simulated drought stress using 13% PEG-6000, which differs from the natural drought stress environment in the field. In addition, the drought stress duration of this study is relatively short and cannot reflect the response of the three species under long-term drought stress. Therefore, it is necessary to conduct long-term drought stress experiments on the three species in the field.

## 4. Materials and Methods

### 4.1. Plant Materials

Plant materials of *S. trilobata*, *S. calendulacea*, and their hybrid species were collected from the South China Botanical Garden, which is affiliated with the Chinese Academy of Sciences in Guangzhou, China. Stem segments (about 120 per species) of the three species with leaves removed were used for the asexual reproduction of the plant materials. Each stem segment was about 15 cm long and contained at least two stem nodes. The stem segments were placed in a beaker containing tap water, and the water surface in the beaker was higher than the lower stem nodes. The stem segments were cultivated in an incubator with a temperature, photoperiod, and light intensity of 25 °C, 14/10 h, and 100 μmol m^−2^ s^−1^, respectively. The plantlets were planted in a Hogland nutrient solution after two weeks of cultivation. When the plants grew 5–6 pairs of leaves, 13% PEG-6000 solution was used for drought stress experiments. In a non-transparent plastic pot containing 13% PEG-6000 solution (25 cm in height, 23 cm in lower diameter, and 26 cm in upper diameter), 15 plants of each species were planted. The roots of the three species were submerged in the 13% PEG-6000 solution. There were 5 (pots) replicates per treatment.

### 4.2. Relative Water Content of Leaves

One plant per pot (5 replicates) was selected, and two leaves (third and fourth leaf positions) were taken from each plant. The leaves were immediately weighed (*A*1) after being cut off from the stem. Then, the leaves were submerged in water for 24 h and weighed again (*A*2). Finally, the leaves were weighed (*A*3) a third time after being dried at 110 °C for 30 min and 70 °C for 24 h. Relative water content was calculated according to Zhang et al. [40]. Relative water content (%) = (*A*1 − *A*3)/(*A*2 − *A*3) × 100%.

### 4.3. Abscisic Acid, Proline, and Soluble Sugar

One plant per pot (5 replicates) was selected, and the leaves at the third and fourth leaf positions were examined to determine the abscisic acid (ABA) content using an ELISA kit (Zike, Shenzhen, China). First, 0.2 g of fresh leaves was accurately weighed and ground in an ice-water bath with 1 mL of 0.05 M phosphate buffer (pH = 7.4). A 2.0 mL centrifuge tube containing 0.5 mL of phosphate buffer was used to collect the ground homogenate. After standing at 4 °C for 2 h, the ABA content was determined according to the manufacturer’s instructions.

One plant per pot (5 replicates) was selected, and the leaves at the third and fourth leaf positions were examined to determine the proline content, according to Zhang et al. [40]. First, 0.25 g of fresh leaves was accurately weighed, and the leaves were ground with 9 mL of 80% ethanol. A 15 mL centrifuge tube containing 0.02 g of active carbon was used to collect the ground homogenate. After standing for 1 h with no light, the ground homogenate was filtered and collected. Standard curves were created using different concentrations of proline, and the proline content was calculated via the standard curves.

One plant per pot (5 replicates) was selected, and the leaves at the second to sixth leaf positions were examined to determine the soluble sugar content, according to Zhang et al. [40]. The leaves were cut from the stem and washed with deionized water. The leaves were dried at 110 °C for 30 min and then at 70 °C until they reached a constant weight. The dry leaves were ground into a powder and sieved with a sieve (0.425 mm). A centrifuge tube (15 mL) containing 8 mL of distilled water was used to collect 8 mg of the sieved sample. The centrifuge tube containing the sample was subjected to an 80 °C water bath for 1 h. After adding 30 mg of activated carbon and mixing well, the centrifuge tube was subjected to an 80 °C water bath for 30 min. After filtration, 10 mL of anthrone sulfuric acid and 1 mL of filtrate were mixed in a 15 mL centrifuge tube. The centrifuge tube containing the mixed liquid was subjected to a 90 °C water bath for 15 min and then cooled. The absorbance was determined at 620 nm. Standard curves were created using different concentrations of glucose, and then the soluble sugar content was calculated.

### 4.4. Enzyme Activity

One plant per pot (5 replicates) was selected, and the leaves at the third and fourth leaf positions were examined to detect the enzyme activity. First, 0.15 g of fresh leaves was accurately weighed and ground in an ice-water bath with 1.5 mL of 0.05 M phosphate buffer containing 0.1% Triton, 0.1 M ethylene diamine tetraacetic acid (EDTA), and 2% polyvinyl pyrrolidone (PVP) (pH = 7.8). The ground homogenate was collected in a 2.0 mL centrifuge tube, and then the centrifuge tube containing the ground homogenate was centrifuged at 4 °C for 15 min at 12,000 *g*. The catalase (CAT), peroxidase (POD), and superoxide dismutase (SOD) activities were determined according to Cai et al. [41].

### 4.5. Malondialdehyde, Hydrogen Peroxide, and Superoxide Anion

One plant per pot (5 replicates) was selected, and the leaves at the third and fourth leaf positions were examined to determine the malondialdehyde (MDA) content, according to Sun et al. [23]. First, 0.15 g of fresh leaves was accurately weighed and ground with 10% trichloroacetic acid (2 mL). A 5.0 mL centrifuge tube was used to collect the ground homogenate, and then it was centrifuged at 4 °C for 15 min at 4000× *g*. A 10 mL centrifuge tube containing 1 mL of 0.67% 2-thiobarbituric acid was used to collect 1 mL of supernatant, and then the centrifuge tube containing 2 mL of the mixed liquid was boiled for 20 min. The absorbance was determined at 600, 532, and 450 nm.

3,3′-diaminobenzidine (DAB) was used to determine hydrogen peroxide, and superoxide anion was determined by nitroblue tetrazolium (NBT), according to Liu et al. [42]. The leaves were completely submerged in 0.5 mg mL^−1^ of DAB dissolved with 0.05 M phosphate solution (pH = 7.0) for 4 h in the dark after being vacuumized three times, and then 80% acetone was used for decolorization. The hydrogen peroxide showed brown matter in the leaves. The leaves were completely submerged in 1 mg mL^−1^ of NBT dissolved with 0.05 M phosphate solution (pH = 6.4) for 4 h in the dark after being vacuumized three times, and then 80% acetone was used for decolorization. The superoxide anion showed a blue substance in the leaves.

### 4.6. Gas Exchange and Fluorescence Parameters

One plant per pot (5 replicates) was selected, and the leaves at the third and fourth leaf positions were examined to determine the gas exchange and fluorescence parameters, according to Zhang et al. [43], with an LI-6800 (LI-COR, Lincoln, NE, USA). The light intensity in the detection chamber was 900 μmol m^−2^ s^−1^, the ratio of red to blue light was 9:1, and the CO_2_ concentration was 400 μmol mol^−1^. The net photosynthetic rate (P_n_), transpiration rate (T_r_), stomatal conductance (G_s_), and intercellular CO_2_ content (C_i_) were recorded after the value became relatively stable. The chlorophyll fluorescence parameters of maximum photochemical efficiency (F_v_/F_m_), actual photochemical efficiency (yield), electron transport rate (ETR), and non-photochemical quenching (NPQ) were measured using a chlorophyll fluorescence imaging system (Technologica, Colchester, UK), according to Baker [44]. The leaves were dark-adapted for 30 min. The minimum fluorescence (F_o_) and the maximum fluorescence (F_m_) of the dark-adapted leaves were measured using a 6000 μmol m^−2^ s^−1^ saturating pulse. F_v_/F_m_ = (F_m_ − F_o_)/F_m_. The actual fluorescence (F′) and the maximum fluorescence (F_m_′) of the leaves exposed to light (PPFD = 900 μmol m^−2^ s^−1^) for 5 min were measured. Yield = (F_m_′ − F′)/F_m_′. ETR = Yield × PPFD × 0.85 × 0.5, where the coefficient 0.85 was the leaf absorptance and the coefficient 0.5 indicated that the absorbed PPFD was equally allocated between PSI and PSII. NPQ = (F_m_/F_m_′) − 1.

### 4.7. Stomatal Observations

The leaves at the fourth leaf position were examined for stomatal characteristics. The leaves were cut into 3 × 3 mm fragments and then collected in a centrifuge tube containing 3 mL fixing solution (2% polyoxymethylene and 2.5% glutaraldehyde). Different concentrations of ethanol were used for fragment dehydration after the fragments were fixed for more than 15 h at 4 °C. A 30 nm gold layer was sprayed after the dehydrated fragments dried [43]. Scanning electron microscopy (Q25, FEI, Hillsboro, OR, USA) was used to record stomatal size.

### 4.8. Gene Expression Analysis

One plant per pot (5 replicates) was selected, and gene expression was detected in the leaves at the second leaf position. Gene expression analysis was conducted according to Zhang et al. [24]. Total RNA from the leaf samples was extracted using TRIzol reagent (Invitrogen, Waltham, MA, USA) according to the manufacturer’s instructions. Complementary DNA was synthesized using TopScript™ RT DryMIX (dT18) (Enzynomic, Daejeon, Republic of Korea) according to the manufacturer’s instructions. Quantitative reverse transcription polymerase chain reaction analysis was performed using a SYBR Premix Ex Taq™ II Kit (Takara, Tokyo, Japan) in conjunction with a Bio-Rad CFX96 Real-Time PCR System (CFX96, Bio-Rad, Hercules, CA, USA). *GAPDH* was used as a reference gene, the primer pairs for which were 5′-GGCTCGACTCGGCATATTCT-3′ (forward) and 5′-CGGCTGCCTTTGGTCTATGT-3′ (reverse). The primer pairs were 5′-TAGCCCAACAACACTTCACTCA-3′ (forward) and 5′-AGCCACCAACACCCTTATGT-3′ (reverse) for *ABA1*, and 5′-GGCATTGGACGCGATTGAAG-3′ (forward) and 5′-TCGAACAACGGGTTAGCTCC-3′ (reverse) for *NCED*. Analysis of the genes’ relative expression levels was conducted according to Livak and Schmittgen [45].

### 4.9. Statistical Analysis

One-way analysis of variance (ANOVA) and Duncan’s post hoc test were used for statistical significance analysis using SPSS Statistics 19.0 (IBM, Armonk, NY, USA) at *p* < 0.05. SigmaPlot 12.5 (Systat Software Inc., San Jose, CA, USA) was used to plot the data.

## 5. Conclusions

The tolerance of the hybrid to drought stress was weaker than that of the invasive species *S. trilobata* but stronger than that of the native species *S. calendulacea*. Under drought stress, the invasive species *S. trilobata* rapidly synthesized more ABA to reduce the leaf stomata opening and water loss. The water loss rate in the hybrid leaves was slower than that in the native species *S. calendulacea*, and it was the slowest in the invasive species *S. trilobata*. The relative water content was 61% in the hybrid, 54% in *S. calendulacea*, and 67% in *S. trilobata*, and it decreased by 33%, 41%, and 28%, respectively, at 8 h under drought stress. The proline content was 23.6 μg g^−1^ in *S. calendulacea*, 27.9 μg g^−1^ in *S. trilobata*, and 25.5 μg g^−1^ in the hybrid at 8 h. The soluble sugar content was 4.5 mg g^−1^ in *S. calendulacea*, 5.3 mg g^−1^ in *S. trilobata*, and 5.1 mg g^−1^ in the hybrid at 8 h. The antioxidant capacity was the strongest in *S. trilobata*, while it was the weakest in *S. calendulacea*. The MDA content was 6.9 μg g^−1^ in *S. calendulacea*, 4.3 μg g^−1^ in *S. trilobata*, and 5.2 μg g^−1^ in the hybrid at 8 h. The MDA content was the highest in *S. calendulacea* and the lowest in *S. trilobata*, and the hybrid showed intermediate results between its two parents. The F_v_/F_m_ value was 7.1 in *S. calendulacea*, 7.5 in *S. trilobata*, and 7.4 in the hybrid at 8 h. The drought resistance of the hybrid did not surpass that of its invasive parent, but it was stronger than that of its native parent, *S. calendulacea*. However, the molecular regulatory mechanisms of the hybrid and its parents in response to drought stress are still unclear and require further research.

## Data Availability

Data are available in the article.
